# Emotional Bias Modification for Individuals With Attention Deficit Hyperactivity Disorder: Protocol for a Co-Design Study

**DOI:** 10.2196/24078

**Published:** 2020-12-23

**Authors:** Melvyn Zhang, Ranganath Vallabhajosyula, Daniel Fung

**Affiliations:** 1 Family Medicine and Primary Care Lee Kong Chian School of Medicine Nanyang Technological University Singapore Singapore; 2 Anatomy Lee Kong Chian School of Medicine Nanyang Technological University Singapore Singapore; 3 Department of Developmental Psychiatry Institute of Mental Health Singapore Singapore

**Keywords:** emotional bias, cognitive biases, ADHD, child psychiatry

## Abstract

**Background:**

Attention deficit hyperactivity disorder (ADHD) is a common neurodevelopmental disorder with a worldwide prevalence rate of 5%. Individuals with ADHD often tend to have difficulties with emotional regulation. The advances in experimental psychology have led to the discovery of emotional biases. Targeting emotional biases could potentially help improve the core symptoms of irritability and short-temperedness among these individuals. Emotional biases refer to the preferential allocation of attention toward emotional stimuli. A recent study reported the presence of emotional biases among individuals with ADHD when they compared individuals with ADHD with those without. Gamification technologies have been explored to help diminish the repetitiveness of the task and increase the intrinsic motivation to train. These inconsistent findings of the impact of gaming on the effectiveness of mobile interventions call for further work to better understand the needs of patients (users) and health care professionals.

**Objective:**

The aim of this research study is to collate health care professionals’ perspectives on the limitations of the existing task, and to determine if gamification elements could be incorporated, to refine the conventional intervention.

**Methods:**

A qualitative research approach, that of a focus group, will be used. Health care professionals from the Department of Development Psychiatry, Institute of Mental Health, Singapore will be invited to participate in this qualitative research. During the focus group, participants are to comment on the limitations of the existing emotional bias intervention; recommend strategies to improve the intervention; and provide their perspectives pertaining to the use of gamification to improve the intervention.

**Results:**

We expect that the study will be completed in 12 months from the publication of this protocol.

**Conclusions:**

To our best knowledge, this is perhaps one of the only few studies that have attempted to explore emotional biases among adolescents with ADHD.

**International Registered Report Identifier (IRRID):**

PRR1-10.2196/24078

## Introduction

Attention deficit hyperactivity disorder (ADHD) is a common neurodevelopmental disorder with a worldwide prevalence rate of 5% [1]. Individuals afflicted with ADHD typically have a constellation of symptoms, characterized by that of hyperactivity, impulsivity, and inattention. The presence of these symptoms often leads to significant psychosocial impairments, for example, that of academic achievements. Individuals with ADHD often tend to have difficulties with emotional regulation too [2]. Because of their inherent difficulties with emotional regulation, these individuals tend to be short-tempered and irritable. The advances in experimental psychology have led to the discovery of emotional biases. Targeting emotional biases could potentially help improve the core symptoms of irritability and short-temperedness among these individuals. Emotional biases refer to the preferential allocation of attention toward emotional stimuli [2]. Emotional biases are most found among individuals who have the combined subtype of ADHD. Children with ADHD-C typically have difficulties not only in emotional processing, but also in comprehension of others’ emotional state, recognition of facial emotions, matching emotional stories, and orientating toward emotional cues [2]. The theoretical approach suggests that these individuals typically have altered top–down processes that are typically responsible for executive planning, inhibition, and cognitive control [2].

The Stroop task has most commonly been used for the assessment and modification of emotional biases and biases related to interference control. In the Stroop task, participants are required to name the colors of the words, while ignoring the semantics of the word. There are limitations to the standard Stroop task, as very often the stimuli are not relevant to individuals with ADHD, and this might affect the assessment for and the modification of such biases. The Dot-probe or visual probe task is an alternative paradigm that was first described by Macleod et al [3]. In the Dot-probe task, participants are presented with 2 stimuli on a screen simultaneously. For individuals with ADHD, these stimuli are usually associated with emotional valence. For example, one of the stimuli might be that of anger expression, whereas the corresponding stimulus is that of a neutral expression. The stimulus would then disappear, and the participant must indicate the position of the probe that comes on. The reaction time that the participant takes to indicate a response following the disappearance of the stimulus is used in the computation of the baseline biases, and in the estimation of how much the bias has been modified. It is expected that individuals with ADHD would react faster when probes replace a stimulus with a positive emotional valence, as compared to probes that replace a stimulus with a negative emotional valence.

The effectiveness of cognitive biases has been well investigated especially among children and adolescents. Krebs et al [4] examined in their review how modification of negative interpretative biases could negate anxiety symptoms among children and adolescents. They identified a total of 26 studies, with each study including individuals up to the age of 18 and reported that cognitive bias modification had moderate effects in reducing negative and positive interpretative biases (Hedges g was 0.70 and 0.52, respectively) [4]. They also reported that bias modification resulted in a small, yet significant reduction of anxiety, with an effect size of 0.17 (Hedges g) [4]. Others have also investigated the utility of cognitive bias modification for hostile interpretative biases [5], and found successful effects of bias modification on hostility. With regard to emotional bias, Cremone et al [6] in their recent study reported the presence of emotional biases among children with ADHD when they compared children with ADHD with those without. In their study, they found that emotional biases were mediated by factors such as the amount of sleep children had. Other studies [7,8] have reported the presence of emotional biases/interference control issues among children with ADHD.

One of the challenges inherent in cognitive bias assessment and modification interventions relates to the laborious nature of these interventions, given that numerous repeats of the trials are required to be undertaken. Serious games and gamification technologies have been applied for mental health interventions. In a previous review, Fleming et al [9] reported the 6 major types of applied games for mental health: exergames, virtual reality, cognitive behavioral therapy (CBT)–based serious games, CBT gamification, biofeedback, and entertainment computer games for mental health, with each having a variable of increasing engagement and some rationalizing the serious purpose of engagement features. Some examples to indicate the role of games in mental health are “SPARX,” an avatar-based video game for CBT, and “JOURNEY,” another video gaming tool to support adolescents depression, which are found have a significant impact in decreasing the adolescent depressive symptoms [9]. Some of the gaming interventions have been evaluated which highlighted that serious games could help improve neuropsychological rehabilitation [10] and that gamification helps improve engagement in the short and long term, promote self-empowerment, and improve the existing skills [11]. The systematic review by Lau et al [12] indicated that serious games could help improve psychiatric symptoms with an effect size of 0.55. There is thus a possibility in considering such technologies for emotional biases intervention, given that such interventions are to be catered for a child and adolescent population. Gamification technologies might also help diminish the repetitiveness of the task and increase the intrinsic motivation to train. These inconsistent findings of the impact of gaming on the effectiveness of mobile interventions call for further work to better understand the needs of patients (users) and health care professionals. Most of these prior interventions have been developed by academics or software developers. The study by Hopia et al [13] reported the perceptions of the end users such as health care professionals and mental health service users on the usage of gamified interventions. Although the views in the study indicated the potential usability of these interventions, these were more generalized. As the interventions could vary according to the type and nature of the specific disorder, there is a need to explore the perceptions of stakeholders belonging to the respective specialties. With the increased recognition of participatory action design research in the recent years [14], such methods could be used to improve existing interventions to suit the needs of patients, and to address existing limitations in various interventions.

The aim of this research study is to collate health care professionals’ perspectives on the limitations of the existing task, and to determine if gamification elements (Table 1) could be incorporated, to refine the conventional intervention. By undertaking this research, we seek to answer the following research questions: (1) What are the perspectives of health care professionals on existing gaming interventions for ADHD; (2) What are the perspectives of health care professionals on a conventional emotional bias modification task?; (3) Would gamification strategies be appropriate, and which gaming strategies are deemed to be the most appropriate? We have decided to sample health care professionals first, to better the existing conventional emotional bias modification paradigm.

**Table 1:** Overview of gamification approaches [15].

Gaming approach		Description
**Economic gamification techniques**		
	Marketplace and economies	Providing gamers with a virtual currency that allows them to deal in the game.
	Digital rewards	These include badges, game currency, game points, virtual goods, and powers or abilities.
	Real-world prizes	Provides gamers with options to exchange in-game credits for real-world prizes such as vouchers or other forms of goods and services.
**Social gamification techniques**		
	Avatar	Allows individuals to choose a virtual character to represent oneself.
	Agent	A virtual character that guides or provides instructions to the user.
	Competition	Allows individuals to compete with other players or with each other.
	Teams	Game that involves several individual players, allowing them to interact and form relationships.
	Parallel communication systems	Allows individuals to communicate with one another.
	Social pressure	Ability of game to pressurize individuals to perform in a certain task, so that he or she will be invited to subsequent events.
**Performance oriented**		
	Feedback	Spoken, visual, or auditory feedback about user’s performance.
	Levels	Information on the stage of a game one has attained.
	Secondary game objectives	Secondary goals that reward the player upon completion.
	Ranks of achievement	Measurement of character development.
	Leaderboards	Allows for comparisons with other players.
	Time pressure	Predetermined time limits for task completion.
**Embedding focused**		
	Narrative context	A storyboard or stories that guide the development of the character.
	3D environment	3D models of objects that parallel the real world.

## Methods

### Study Design

For the purposes of this research, a qualitative research approach (focus group) will be used. We will also adopt some principles from participatory action research, which is a form of research that uses systematic inquiry, along with the participation of the relevant stakeholders. This helps to refine educational processes or effecting a social intervention.

### Study Setting

We seek to invite health care professionals from the Department of Development Psychiatry, Institute of Mental Health, Singapore to participate in this qualitative research. The purpose of including health care professionals is mainly due to their expertise in the treatment of children/adolescents with ADHD. A diverse group of participants will be recruited, as the group comprises not only psychiatrists, but also psychologists and occupational therapists. It is expected that psychiatrists will comment on how this intervention will complement their existing pharmacological interventions, and psychologists and occupational therapists will comment on how this intervention will supplement or complement existing psychological approaches.

### Sample Size

A total of 8 participants will be recruited for the study. The participants will include 3 psychiatrists, 3 psychologists, and 2 therapists for the focus group. To recruit the participants, the principal investigators (MZ and RV) will approach the head of the department to seek approval if an email could be circulated among the department to inform about the study. Participants willing to voluntary participate in the study could reply to the principal investigators’ email. The principal investigators will liaise with the participants regarding the date and time of the study.

### Details of the Planned Focus Group

All participants who provide their consent for the study will be asked to complete a baseline demographic questionnaire. This questionnaire will collate information about their age, gender, and their years of experience in child psychiatry/treatment of children with psychiatric disorders. Following the completion of the questionnaire, the study team members will email all the participants and identify a common date and time in which the focus group could be conducted. The duration of the focus group discussion is expected to last between 1.5 and 2 hours.

Both principal investigators will facilitate the focus group and record field notes. At the start of the focus group discussion, participants will be informed about the rationale of the project as well as the specific objectives of the sessions. Participants will be reassured that their comments are confidential, and will be told that their comments will be audio-recorded. Participants will be shown some examples of existing gaming interventions in the published literature for the treatment of ADHD symptoms. Participants are asked to comment on the advantages and the limitations of these interventions. In the event that participants do not feel comfortable in commenting, they are also provided with written materials, for them to note down their comments. Following the completion of this phase, participants are shown examples of cognitive bias modification interventions, and specifically, an example of an emotional bias modification intervention. Participants are asked to comment on the benefits of such a form of intervention, identify limitations, and suggest possible methods to overcome the limitations. Following this, participants are shown a list of gamification techniques, and the facilitators (MZ and RV) will explain to them more about the specific gamification techniques. Participants will be asked if the inclusion of such techniques would be appropriate for the existing application.

### Data Analyses

The workshops will be audio-recorded and transcribed verbatim subsequently. The principal investigator MZ will listen to the audio recordings of the workshop and develop a coding frame. Two separate researchers will also review and code the transcripts. The codes will then be reorganized into themes. NVivo version 12.0 will be used in the analysis.

### Data Management and Monitoring

No participant-related identifiers will be captured on the hard copy questionnaires. All the completed hard copy forms and the informed consent forms will be stored in a secured facility, under lock and key. The audio recordings of the workshop will be transferred onto a local secured computer for storage and the recording will also be removed from the recording device. The password of the local computer will be changed frequently, and only the principal investigator (MZ) will have access to the local computer. All the records and audio recordings will be kept for 6 years after the completion of the study.

### Adverse Events

Any adverse events that occur during the conduct of the study will be reported to the domain-specific research board according to the local institutional policy.

### Ethical Approval

The study has been approved by the Ethical Review Board of Nanyang Technological University Singapore (IRB-2020-03-058). The data obtained could be potentially used to develop the novel prototype that suits the needs of the stakeholders.

## Results

We expect that the study will be completed in 12 months from the publication of this protocol. The findings arising from this study will be published in academic journals, and presented at both local and international conferences.

## Discussion

To our best knowledge, this is perhaps one of the only few studies that have attempted to explore emotional biases among individuals with ADHD. The utilization of participatory action design research, in the design and conceptualization of a co-designed application, is novel, and helps to ensure that the conceptualized application is based on evidence. While the perspectives of health care professionals are critical in bettering the existing conventional task, and ensuring that it maintains the evidence base, it is nevertheless important to seek out the perspectives of the service user/individuals with ADHD. We intend to undertake this as soon as we have completed the existing study.

## Abbreviations

ADHD: attention deficit hyperactivity disorder
CBT: cognitive behavioral therapy
